# Oral microbial communities in children, caregivers, and associations with salivary biomeasures and environmental tobacco smoke exposure

**DOI:** 10.1128/msystems.00036-23

**Published:** 2023-06-20

**Authors:** Jason A. Rothman, Jenna L. Riis, Katrina R. Hamilton, Clancy Blair, Douglas A. Granger, Katrine L. Whiteson

**Affiliations:** 1 Department of Molecular Biology and Biochemistry, University of California, Irvine, California, USA; 2 Institute for Interdisciplinary Salivary Bioscience Research, University of California, Irvine, California, USA; 3 Department of Psychological Science, University of California, Irvine, California, USA; 4 Department of Psychiatry and Behavioral Sciences, Johns Hopkins University School of Medicine, Baltimore, Maryland, USA; 5 Department of Population Health, New York University, New York, New York, USA; 6 Department of Applied Psychology, New York University, New York, New York, USA; 7 Department of Acute and Chronic Care, Johns Hopkins University School of Nursing, Baltimore, Maryland, USA; 8 Department of Pediatrics, Johns Hopkins University School of Medicine, Baltimore, Maryland, USA; 9 Salivary Bioscience Laboratory, University of Nebraska, Lincoln, Nebraska, USA; 10 Department of Psychology, University of Nebraska, Lincoln, Nebraska, USA; Northern Arizona University, Flagstaff, Arizona, USA

**Keywords:** microbiome, oral microbiome, social ecology, 16S rRNA sequencing

## Abstract

Human oral microbial communities are diverse, with implications for oral and systemic health. Oral microbial communities change over time; thus, it is important to understand how healthy versus dysbiotic oral microbiomes differ, especially within and between families. There is also a need to understand how the oral microbiome composition is changed within an individual including by factors such as environmental tobacco smoke (ETS) exposure, metabolic regulation, inflammation, and antioxidant potential. Using archived saliva samples collected from caregivers and children during a 90-month follow-up assessment in a longitudinal study of child development in the context of rural poverty, we used 16S rRNA gene sequencing to determine the salivary microbiome. A total of 724 saliva samples were available, 448 of which were from caregiver/child dyads, an additional 70 from children and 206 from adults. We compared children’s and caregivers’ oral microbiomes, performed “stomatotype” analyses, and examined microbial relations with concentrations of salivary markers associated with ETS exposure, metabolic regulation, inflammation, and antioxidant potential (i.e., salivary cotinine, adiponectin, C-reactive protein, and uric acid) assayed from the same biospecimens. Our results indicate that children and caregivers share much of their oral microbiome diversity, but there are distinct differences. Microbiomes from intrafamily individuals are more similar than microbiomes from nonfamily individuals, with child/caregiver dyad explaining 52% of overall microbial variation. Notably, children harbor fewer potential pathogens than caregivers, and participants’ microbiomes clustered into two groups, with major differences being driven by *Streptococcus* spp. Differences in salivary microbiome composition associated with ETS exposure, and taxa associated with salivary analytes representing potential associations between antioxidant potential, metabolic regulation, and the oral microbiome.

**IMPORTANCE:**

The human oral cavity is a multi-environment habitat that harbors a diversity of microorganisms. This oral microbiome is often transmitted between cohabitating individuals, which may associate oral and systemic health within family members. Furthermore, family social ecology plays a significant role in childhood development, which may be associated with lifelong health outcomes. In this study, we collected saliva from children and their caregivers and used 16S rRNA gene sequencing to characterize their oral microbiomes. We also analyzed salivary biomeasures of environmental tobacco smoke exposure, metabolic regulation, inflammation, and antioxidant potential. We show there are differences in individuals’ oral microbiomes mainly due to *Streptococcus* spp. that family members share much of their microbes, and several bacterial taxa associate with the selected salivary biomeasures. Our results suggest there are large-scale oral microbiome patterns, and there are likely relationships between oral microbiomes and the social ecology of families.

## INTRODUCTION

The human oral cavity hosts a dense and diverse community of microorganisms that is associated with health and a variety of diseases (1 - 4). A typical human oral microbiome comprises hundreds of bacterial taxa, ranging from commensals to pathogenic species that cause caries and infections such as periodontitis and gingivitis (3, 4). Aside from directly causing infections, there is growing evidence that the oral microbiome is involved in systemic disease, including diabetes, cardiovascular disease, and oral cancer (4 - 7). In this study, we focus on the similarities and differences in oral microbial taxa between children and their caregivers, and the associations between children’s oral microbial community composition and measures of environmental tobacco smoke (ETS) exposure, inflammation, and oxidative stress. There is an intricate relationship between the social environment, exposome, host physiology and microbes of the oral cavity, and understanding microbial population dynamics can aid in disentangling the oral microbiome’s role in human health and development.

The oral microbiome is one of the most diverse microbial communities found in humans—second only to the gut in overall bacterial diversity—and represents a unique multi-habitat environment that is exposed to both the outside and inside of the body (3). Generally, the oral microbiome consists of taxa in the phyla *Firmicutes*, *Fusobacteria*, *Proteobacteria*, *Actinobacteria*, *Bacteroidetes*, and others, while at the strain level, individuals have personalized bacterial profiles (1, 2). Interestingly, the oral microbiome is acquired over time (8 - 10), and taxa are often shared between caregiver and child resulting in microbial communities among family members being similar (11, 12). Human microbiomes are thought to be transmitted through contact with the mother during birth (13), and augmented by subsequent environmental exposures and close social contact. While intrafamily interactions are important for microbial transmission, previous large-scale studies have shown that people also tend to share community types regardless of family status, and that these groupings may have implications for oral health and overall wellbeing (14, 15).

Several factors influence the composition of human oral fluids, including nutrition, disease, medication use, and the presence of microbes (3). As a complex biofluid, saliva contains thousands of metabolites, chemical compounds and proteins (16, 17). Oral bacteria live in contact with oral fluid—and receive nutrition from salivary components—factors that affect salivary production and composition are important in understanding host/microbe relationships (3). For instance, research has been conducted on the interactions between oral microbes and host health measured through inflammation and inflammatory molecules (18). Likewise, smoking has been shown to alter the composition of oral taxa (19) and increase dental caries (20). Furthermore, there is evidence that the oral microbiome produces small molecules that the host may uptake, and some microbial taxa may indicate an individual’s overall health (21). As part of an exploratory study into the oral microbiome and host physiology, we associated oral taxa with the following biomeasures: C-reactive protein (CRP), a molecule that corresponds to systemic inflammation and has been implicated in periodontitis (22), adiponectin, a protein involved in metabolic regulation and associated with oral inflammation (23, 24), cotinine, a nicotine metabolite and indicator of environmental tobacco smoke exposure (25), and uric acid, the end product of the purinergic system involved in stress response and antioxidant potential (26).

In the context of a large-scale, prospective, longitudinal study of child development and the ecology of the home and family environment known as the Family Life Project (FLP), we investigated the relationships between the human oral microbiome and the similarities of microbiome composition between children and their caregivers. We asked several questions: First, does oral microbiome composition differ between children and adults, and do family members share a greater proportion of their oral microbes than nonfamily members? Second, are any members of the oral microbiome associated with biomeasures related to inflammation, metabolic regulation, ETS exposure, or antioxidant potential? Lastly, are there bacterial taxa common across people, and are there underlying patterns that may indicate oral dysbiosis?

## MATERIALS AND METHODS

### Participants and procedures in study

The FLP is a prospective longitudinal study of families residing in six predominantly low-income and nonurban counties in central Pennsylvania or eastern North Carolina. Complex sampling procedures were used to recruit a representative sample of 1,292 children whose families resided in the target communities at the time the mothers gave birth. Participants were oversampled for poverty in both states, and African Americans were oversampled in North Carolina. Detailed descriptions of the sampling and recruitment procedures are available in (27). Briefly, families with a child born between September 2003 and August 2004 were recruited from hospitals at the time of birth, and participating families have completed regular interval follow-up assessments. The current analyses focused on a subset of data collected at the 90 month follow-up, where at home, children and their primary caregivers provided saliva samples. For the purposes of this study, the cohoused child and adult who provided parental care (“caregiver”) were paired together where possible. Of the 224 dyads, the vast majority of parental caregivers were biological parents (*N* = 221), while 1 dyad contained an adoptive parent, and 2 dyads contained a foster parent. These saliva samples were assayed and archived in −80°C freezers. Archived biospecimens with adequate saliva remaining for microbiome analysis were examined in this study (child *N* for this microbiome subsample = 294, male = 164, female = 130; age 79 to 100 months, average = 87 months); caregiver *N* for this microbiome subsample= 430, females = 429 females, males = 1). Caregivers also reported on their smoking status during the study appointment. Procedures for this study were run under the IRB of the University of North Carolina (IRB # 07-0646 and 16-2751) and New York University (IRB # IRB-FY2017-69) using deidentified data. Sample IDs were further randomized prior to analysis and reporting.

### Biospecimen collection and determination of salivary biomeasures

Following Granger and colleagues (28), whole saliva was collected from children and caregivers by passive drool. Samples were immediately frozen, then transported to the Institute for Interdisciplinary Salivary Bioscience Research (IISBR) at the University of California, Irvine for assay and archiving. On the day of assay, samples were thawed and centrifuged to precipitate mucins. All samples were assayed in duplicate for each biomeasure.

#### Adiponectin

Salivary adiponectin was determined using the Human Adiponectin Meso Scale Discovery (MSD) Assay kit (Meso Scale Diagnostics, Rockville, MD, USA). Samples were diluted fivefold, then assayed following the manufacturer’s supplied protocol using a four-log standard curve. Concentrations were derived using MSD Discovery Workbench software v4.0 (ng/mL) using curve fit models (4-PL with a weighting function option of 1/y^2^). The average intra-assay coefficient of variation (CV) was 3.8%, average inter-assay CV was 2.4%, and the assay range of sensitivity was 0.06 to 1,000 ng/mL.

#### C-reactive protein (CRP)

Salivary CRP was determined using the Human CRP (Vascular Injury Panel 2) V-Plex MSD multi-spot Assay (Meso Scale Diagnostics, Rockville, MD, USA). Samples were diluted fivefold and used a five-log standard curve following the manufacturer’s recommended protocol. MSD Discovery Workbench Software V4.0 was used to determine CRP concentrations (pg/mL) using curve fit models (4-PL with a weighting function option of 1/y^2^). The average intra-assay CV for CRP was 3.07%, average inter-assay CV was 2.4%, and the assay range of sensitivity was 9.9 to 1,010,000 pg/mL.

#### Cotinine

Salivary cotinine was determined using a commercially available, enzyme-linked immunosorbent assay kit (Salimetrics, Carlsbad, CA, USA) following the manufacturer’s protocol. In caregivers, if nicotine use was reported, the saliva sample was prediluted 10-fold before assay. Samples from caregivers not reporting nicotine use and from children were tested neat. Cotinine concentrations were computed from a standard curve generated using a four-parameter nonlinear regression curve fit (Gen5, BioTek, Winooski, VT, USA). Cotinine measurements had an average inter-assay CV of 9.1% and the assay range of sensitivity was 0.15 to 200 ng/mL for neat saliva and 1.5 to 2,000 ng/mL for 10-fold diluted saliva.

#### Uric acid

Salivary uric acid was determined using an enzymatic assay kit following the manufacturer’s protocol (Salimetrics, Carlsbad, CA, USA), and uric acid concentrations were derived in mg/dL with Gen5 software (Gen5, BioTek). Uric acid measurements had an average intra-assay CV of 3.6%, average inter-assay CV of 2.4%, and the assay range of sensitivity was 0.07 to 20 mg/dL.

### Preanalysis of salivary biomeasures

As reported above, each analyte was measured independently and had different limits of detection (LOD) and lower limits of quantification (LLOQ). We removed samples from downstream analyte-specific analyses in cases where the CVs were greater than 15% (*N* = 2 from CRP, *N* = 1 from cotinine, and *N* = 1 from uric acid-specific analyses). We substituted values of 1/2 the LLOQ for each sample under the LLOQ [*N* = 6 for adiponectin, *N* = 27 for CRP, *N* = 129 (*N* = 76 children and *N* = 53 caregivers) for cotinine, and *N* = 28 for uric acid].

### Microbiome sampling procedure and preprocessing

Saliva samples were thawed, mixed at room temperature, aliquoted 200 µL into cryovials, and we shipped the aliquots to the Integrated Microbiome Resource (IMR) at Dalhousie University for DNA extraction, library preparation, and 16S rRNA gene sequencing. We would like to disclose that all the samples were misplaced by Federal Express during shipping and sat at ambient temperature for 1 week in a shipping warehouse before being received by IMR, while other samples never arrived.

### DNA extraction, library preparation, and next-generation sequencing

IMR handled all DNA extraction, library preparation, and MiSeq sequencing of our samples. Briefly, IMR extracted DNA from saliva samples using Qiagen PowerFecal kits (Qiagen, Germantown, MD, USA) with bead-beating, then prepared sequencing libraries targeting the V6-V8 regions of the 16S rRNA gene with an in-house protocol (29). The V6-V8 region was recommended by IMR to reduce the amplification of host mitochondria. IMR quantified libraries and with a Qubit fluorescence reader and by agarose gel electrophoresis. IMR then pooled the libraries and sequenced them on an Illumina MiSeq sequencer using paired-end V3 2 × 300 bp kits.

### 16S rRNA gene sequence library processing

IMR used the QIIME2 (30) pipeline to process the 16S rRNA gene sequences. First, sequences were visualized, sequences and primers and low-quality ends were trimmed off of the reads with QIIME2, reads were joined, then Deblur (31) was used to remove chimeras and bin sequences into Amplicon Sequence Variants (ASVs; DNA sequences that are 100% identical). Once sequences were binned, taxonomy was assigned with the q2-feature-classifier “classify-sklearn” (32) trained to the V6-V8 region of the 16S rRNA gene with the SILVA database (release 132) (33). IMR then filtered out ASVs present at less than 0.01% relative abundance to account for singletons and likely contaminants and generated a final ASV table (includes unique ASV IDs and SILVA taxonomy; Zenodo dataset doi: 10.5281/zenodo.7523443) which we used for all downstream analyses (34).

### Bioinformatics and statistics

We used QIIME2 v2019.7 and the computing environment R (35) to generate Shannon Diversity indices and Bray–Curtis dissimilarities for each sample and calculate taxa relative abundances. We compared the effects of categorical variables on alpha diversity through Kruskal–Wallis tests, and beta diversity using Adonis (nonparametric ANOVA with 999 permutations) on rarefied data with the R package “vegan” (36), and used ANCOM (37) for differential abundance testing of taxa on unrarefied data. We used the R package “MaAsLin2” (38) to assess relationships between adiponectin, CRP, cotinine, uric acid concentrations, and unrarefied relative abundances of bacterial ASVs.

To further investigate the salivary microbiome, we visualized the data with nonmetric multidimensional scaling (NMDS) of the Bray–Curtis dissimilarities using the categorical variables “caregiver/child,” “smoking/nonsmoking” for caregivers, and “< 1 ng/mL or >1 ng/mL” cotinine concentration for children as a conservative estimate for ETS exposure (25), then plotted the ordinations with the R packages “ggplot2” (39) and “patchwork” (40). We also used distance-based Redundancy Analysis (db-RDA) in “vegan” to assess the contributions of continuous variables to microbiome variability and plotted the resulting ordinations with “ggplot2.”

In addition to the above analyses, we performed stomatotype analysis [as in (14, 41)] on ASVs in the paired caregiver/child dyads at greater than 0.01% relative abundance using Jensen–Shannon distances and partitioning around medoid (PAM) clustering with the R packages “ade4” (42) and “cluster” (43) as in (41). PAM clustering is an unsupervised method for determining underlying patterns in microbiome data without including metadata variables and is considered more robust than the related k-means methods (44). Likewise, we used Jensen–Shannon distances as this measure often results in more accurate clustering than Manhattan-based measures (i.e., Bray–Curtis) (45). We estimated optimal cluster number with the Calinski–Harabasz (CH) index. Once we had determined optimal cluster number, we used ANCOM to test individual ASVs for differential abundance between the cluster assignments and plotted a PCoA ordination and boxplots of the data with “ggplot2” and “patchwork.”

## RESULTS

### Overall library statistics

**Fig 1:**
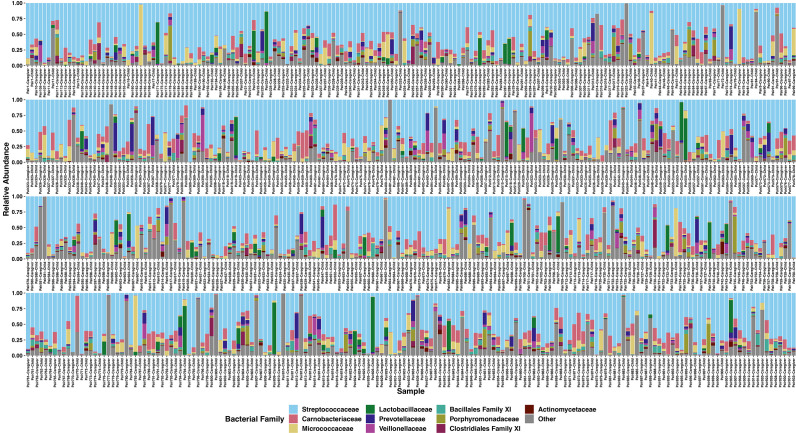
Stacked bar plots of the relative abundances of the 10 most proportionally abundant bacterial families plus all others in children’ and caregivers’ oral microbiomes. Color denotes bacterial family, and labels indicate which dyad and source (child or caregiver) each sample belongs to.

We obtained 17,966,151 16S rRNA gene reads assigned to 6,659 ASVs across 724 samples (*N* = 294 children, *N* = 430 caregivers) with an average of 24,815 reads per sample. Through rarefaction analyses, we determined that we had acceptable coverage at a sequencing depth of 2,000 reads per sample (Fig. S1). Overall, our samples were dominated by few taxa, with the 10 most abundant bacterial families comprising an average relative abundance of 86.9% of overall relative abundance. These families were *Streptococcaceae* (60.7%), *Carnobacteriaceae* (7.6%), *Micrococcaceae* (5.7%), *Lactobacillaceae* (2.7%), *Prevotellaceae* (2.5%), Bacillales Family XI (2.1%), *Veillonellaceae* (2.0%), *Porphyromonadaceae* (1.8%), Clostridiales Family XI (0.9%), and *Actinomycetaceae* (0.8%) (Fig. 1). Within these families, several ASVs were in high relative abundance, with the three most abundant ASVs (all *Streptococcus*) accounting for 55.5% of proportional bacterial abundance. Lastly, 10 ASVs were present in at least 75% of samples: one ASV from each of *Actinomyces*, *Atopobium*, *Gemella*, *Granulicatella*, *Porphyromonas*, *Rothia, and Veillonella*, and three ASVs from *Streptococcus*.

### Stomatotype cluster analyses of caregivers and children

**Fig 2:**
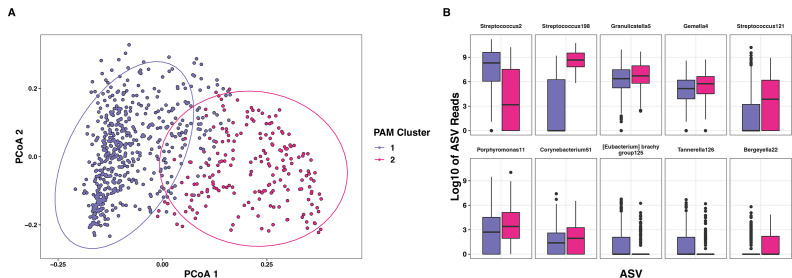
(A) PCoA of Jensen–Shannon distances from the partitioning around medoid (PAM) clustering results of Amplicon Sequence Variants (ASVs) at greater than 0.01% relative abundance in samples, colored by cluster assignment in caregiver/child dyads. (B) Boxplots of the Log_10_ adjusted counts of differentially abundant ASVs between PAM clusters as measured by ANCOM.

Samples from caregivers and children were paired into 224 dyads (*N* = 448 samples). As microbial populations often diverge between groups of people, we analyzed the oral microbiomes of caregiver/child dyads through PAM clustering and observed that oral microbial communities clustered into two major groups, with no metadata category obviously corresponding to cluster assignment (Fig. 2). We then used ANCOM and show that there were 10 significantly differentially abundant ASVs between the PAM clusters (*W* > 134, *P*_adj_ < 0.05, Fig. 2). One cluster was dominated by ASVs within the *Streptococcus mitis* group (“Streptococcus2”; this ASV 100% matches *S. oralis. S. cristatus*, and *S. mitis*) and *Tannerella forsythia* (“Tannerella126”), and the other cluster was mainly comprised of ASVs of *Streptococcus oralis dentisani* (“Streptococcus198”) and *Streptococcus parasanguinis* (“Streptococcus121”). Other bacterial taxa important in cluster assignment were ASVs of *Granulicatella*, *Gemella, Porphyromonas, Corynebacterium, Eubacterium*, and *Bergeyella*, a likely pathogenic genus previously found in oral microbiome studies (2) (Fig. 2).

### Microbial diversity of caregiver/child dyads

**Fig 3:**
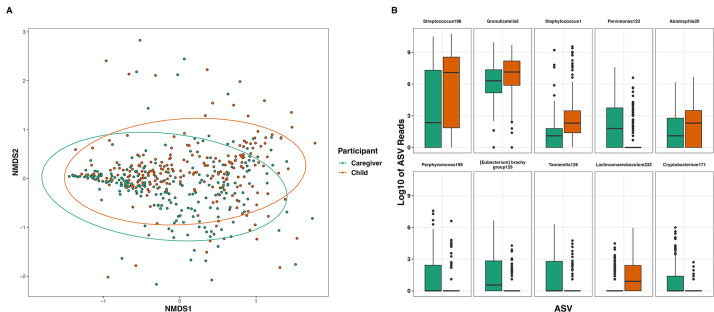
(A) Nonmetric multidimensional scaling (NMDS) plot of the Bray–Curtis dissimilarities of children and caregivers. Microbiomes in children (*N* = 224) were significantly different from microbiomes in caregivers (*N* = 224) (*R^2^* = 0.02, *F* = 10.56, *P* < 0.001), and caregiver/child dyad explained the majority of microbial variation between samples (*R^2^* = 0.52, *F* = 1.13, *P* = 0.003). (B) Boxplots representing significantly different Amplicon Sequence Variants (ASVs) between caregivers and children as tested by ANCOM. Data were Log_10_ transformed for plotting.

We found that the beta diversity of children and caregivers significantly differed (*R^2^* = 0.02, *F* = 10.56, *P* < 0.001), and that caregiver/child dyad explained the majority of variation between our samples (*R^2^* = 0.52, *F* = 1.13, *P* = 0.003) (Fig. 3). Alpha diversity was not significantly different between pairs of caregivers and children comprising the dyads (*H* = 258, *P* = 0.053), or between caregivers and children overall (*H* = 0.001, *P* = 0.98). We then compared bacterial ASVs between caregivers and children and showed that 10 ASVs (*W* > 145, *P*_adj_ < 0.05 for each ASV) were significantly different (Fig. 3).

### Distance-based redundancy analysis of continuous variables on children’s microbiome

**Fig 4:**
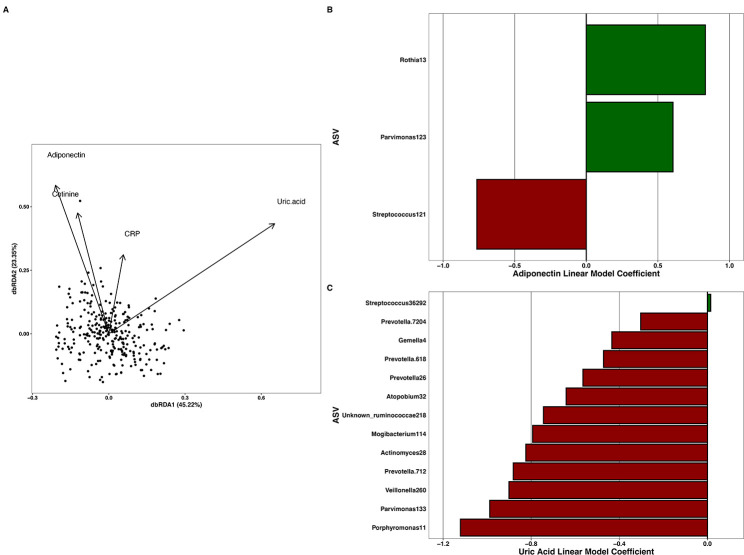
(A) Biplot of a distance-based redundancy analysis on the Bray–Curtis dissimilarities of children who had all biomeasures. In the distance-based Redundancy Analysis (db-RDA) overall, analytes significantly associated with children’s oral microbiomes (*N* = 288, *F* = 1.66, *P* < 0.001), with adiponectin, cotinine, and uric acid (*P* < 0.05 for each) individually significantly associating with the oral microbiomes, while C-reactive protein (CRP) did not (*P* = 0.61, not shown). (B and C) Significant MaAsLin2 associations and linear model coefficients between ASVs from *N* = 294 children at greater than 0.05% relative abundance and (B) adiponectin or (C) uric acid concentrations. As CRP was only associated with one Amplicon Sequence Variant (ASV), those data are not shown here.

In order to compare the association of each biomeasure (adiponectin, CRP, uric acid, and cotinine) of children’s oral microbiomes, we used distance-based Redundancy Analysis (db-RDA) on the Bray–Curtis dissimilarities of samples who had measurements for all biomeasures and oral microbiome (*N* = 288 children). Overall, biomeasures were significantly associated with children’s oral microbiomes but only explained 2.3% of the variation (*F* = 1.66, *P* < 0.001, Fig. 4). We also tested the contribution of each biomeasure independently with PERMANOVA, and found adiponectin (*F* = 1.57, *P* = 0.003), cotinine (*F* = 1.56, *P* = 0.003), and uric acid (*F* = 2.49, *P* < 0.001) were significantly associated with microbiome variation, while CRP was not (*F* = 1.02, *P* = 0.43).

### Associations of the microbiome with adiponectin, uric acid, and CRP

Using the same data in our db-RDA analysis, we included samples from children that we measured concentrations of adiponectin, uric acid, or CRP for and microbiome data and used MaAsLin2 to find associations between each salivary biomeasure and individual microbial ASVs at greater than 0.05% relative abundance. Adiponectin and three ASVs were significantly associated, one ASV was associated with CRP, and 13 ASVs were significantly associated with uric acid (*P*_adj_ < 0.05, Fig. 4).

### Overall associations of ETS exposure with the oral microbiome

We used caregivers’ survey data and children’s salivary cotinine concentrations to determine smoking status and ETS exposure. Because the microbiomes of caregivers and children were significantly different, and caregivers and children likely have different routes of ETS exposure, we analyzed the effects of tobacco smoke exposure on caregivers (*N* = 239) and children (*N* = 281) separately.

**Fig 5:**
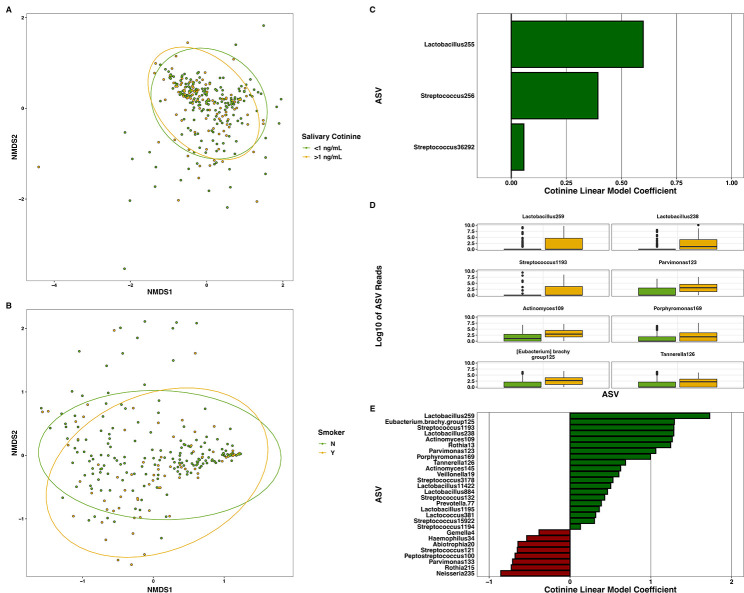
Nonmetric multidimensional scaling (NMDS) plot of the Bray–Curtis dissimilarities between (A) children (*N* = 281) with salivary cotinine levels of <1 ng/mL or >1 ng/mL, and (B) nonsmoking or smoking caregivers (*N* = 239). Exposure to ETS was significantly associated with altered microbiome beta diversity in caregivers (*R^2^* = 0.01, *F* = 2.61, *P* = 0.025) but not children (*R^2^* = 0.005, *F* = 1.34, *P* = 0.17). Panel C and E illustrates statistically significant MaAsLin2 associations of microbes at > 0.01% relative abundance with child and caregiver cotinine concentrations, respectively. Panel D shows boxplots of individual Amplicon Sequence Variants (ASVs) whose abundances significantly differed between caregiver smokers and nonsmokers as tested by ANCOM. Data in panel D were Log_10_ transformed for plotting.

In caregivers, bacterial beta diversity was slightly different in smokers than nonsmokers (*R^2^* = 0.01, *F* = 2.76, *P* = 0.01, Fig. 5), whereas alpha diversity did not significantly differ (*H* = 0.57, *P* = 0.45). We used ANCOM for differential abundance testing and observed significant differences in the relative abundances of eight bacterial ASVs (*W* > 156, *P*_adj_ < 0.05, Fig. 5). Additionally, we analyzed MaAsLin2 associations between cotinine concentrations and individual ASVs and observed that 28 bacterial ASVs were significantly associated with cotinine concentrations (*P*_adj_ < 0.05, Fig. 5).

When comparing the microbiomes from children with salivary cotinine levels < 1 ng/mL to those with levels >1 ng/mL, we found ETS exposure was associated with slightly increased children’s alpha diversity (*H* = 4.5, *P* = 0.035), but not beta diversity (*R^2^* = 0.005, *F* = 1.31, *P* = 0.19), and was not related to any did not affect any bacterial ASV (*P*_adj_ >0.05 for each) through ANCOM testing. When analyzing MaAsLin2 associations, only three ASVs were significantly associated with cotinine concentrations (*P*_adj_ < 0.05, Fig. 5).

## DISCUSSION

Using biological assessments from the large-scale FLP, we explored relations between the human oral microbiome, salivary measures related to metabolic activity, inflammation, ETS exposure, and antioxidant potential, and the similarities of microbiome composition in families. The oral microbiomes of within-family dyads of caregivers and their children explained most of the variation in oral microbiomes, and overall—regardless of dyad—children and adults had slightly different oral microbial communities, which supports previous work (11, 12, 46). We also found that participants clustered together by microbial composition, representing a broad view of bacterial communities between people. This indicates there are likely “stomatotypes” across human oral microbiomes that may be involved in oral health (14, 15). Likewise, we investigated the associations of ETS exposure and oral microbiomes and saw moderate microbial differences in adults who smoke and minor microbial differences in children environmentally exposed to smoke. Finally, through exploratory analyses, we found several associations between microbiome and salivary biomeasures related to metabolism, inflammation, and children’s antioxidant marker uric acid levels, which suggests that levels of these biomeasures may be important in host-microbe interactions.

Overall, children and adults had slightly different oral microbiomes, with this comparison explaining 2% of the variation, and several taxa were differentially abundant between the two groups. For example, children had higher relative abundances of bacteria thought to be associated with good oral health, such as *Streptococcus oralis dentisani* (47) while having lower relative abundances of pathogenic bacteria such as *Tannerella forsythia* (48), *Porphyromonas gingivalis* (49), and *Eubacterium* spp (50). Our findings support the hypothesis that children’s oral microbiomes are populated more by early-colonizing bacteria (9), and that the proportion of disease-associated oral microbes may increase with age (12, 46). Although child and adult oral microbiomes were slightly different overall, we found that family members shared much of their microbial community composition, as child/caregiver dyad pairing explained 52% of the microbial variance—the most substantial association in our data. This finding supports research indicating that intrafamily microbial communities are often similar, and that there is transmission of oral microbes between family members (11, 12) likely due to a shared home environment and similar diets. While ours and others’ research is informative, we were unable to identify taxa to strain-level, and we suggest that future studies do this by using metagenomics to examine the oral microbiome of children and their caregivers. We also recognize that our data are compositional (51), and we may be observing artifacts of next-generation sequencing instead of true differential abundances of taxa.

Through PAM-cluster analyses, we found that the oral bacterial communities of our subjects clustered into two major overlapping groups. The separation of these groups was mainly driven by three *streptococcus* ASVs—and to a lesser extent *Eubacterium, Tannerella, Bergeyella,* or *Porphyromonas*. Notably, subjects in one cluster were dominated by *S. mitis*, while the other had higher abundances of *S. oralis dentisani* and *S. parasanguinis*. While these species are generally health-associated, there is some debate over their potential pathogenicity (52). For example, *S. mitis* group bacteria have been associated with dental caries (53), but are also frequently found in healthy mouths. Similarly, *S. oralis dentisani* inhibits cariogenic *S. mutans* (47), and *S. parasanguinis* antagonizes *Pseudomonas aeruginosa* in cystic fibrosis patients (54). Furthermore, oral bacteria often co-aggregate, which can form microenvironments for competition/cooperation and may cause situational population dynamics to occur (55). Coupled with the difficulty in resolving *Streptococcus* species through 16S rRNA gene sequencing (56), we were unable to fully determine the relationships between the three *S*. *mitis* taxa and suggest that future research investigate the competition/cooperation of these bacteria, especially in the context of oral and systemic health.

Previous large-scale “stomatotype” studies have placed subjects’ oral microbiomes into clusters as well. For instance (14, 14) observed adolescents’ microbial communities being mainly separated into clusters of *Prevotella-Veillonella* or *Neisseria-Haemophilus*. Likewise, Takeshita et al. (15) reported overlap in the clusters of their adults-only study and suggest a continuum of clusters being separated by *Neisseria-Haemophilus*, *Streptococcus-Rothia*, and *Prevotella-Veillonella-Streptococcus*. Our work differs by sampling both children and adults, and observing cluster separation by *Streptococcus* ASVs, and minorly *Eubacterium-Tannerella* or *Bergeyella-Porphyromonas* regardless of subject age. As we sampled both children and adults—and concentrated on moderate/high abundance ASVs instead of genera—we expected to find differences between ours and others’ work. Our study adds to the growing literature of large-scale oral microbiome studies and supports the concept of population-level divisions in oral communities.

We analyzed the relations between ETS exposure and oral microbiomes in both children and caregivers. ETS-exposed adults had higher microbial diversity, which is consistent with other studies (15, 57); and that ETS exposure was very slightly associated with increased microbial diversity in children. Likewise, several ASVs were significantly different between adult smokers and nonsmokers; for example, taxa within the potentially pathogenic genera *Eubacterium* (50) and *Porphyromonas* were in higher proportional abundance in smokers, along with the debatably beneficial species *Lactobacillus fermentum* and *L. gasseri/hominis/johnsonii* (we were unable to resolve this ASV to species) (58, 59). While it is unknown how smoking influences individual species of lactobacilli, our findings are in line with previous studies showing lactobacilli abundances are positively-associated with smoking (57, 60), and that these bacteria may act as opportunists in smokers’ mouths. Although exposure to ETS is associated with poor health outcomes among children, including bacterial infections and dental caries (20, 61), we did not observe major relationships between our index of ETS exposure and children’s oral microbiomes. As adult’s overall oral microbiomes were associated with smoking status, our data support the potential of smoking to cause bacterial dysbiosis, which may lead to worsened infections and inflammation, although this may be confounded by other lifestyle factors that we did not examine in this study (62).

In addition to categorical smoking status, we examined the associations between cotinine concentrations and proportional abundances of individual ASVs in adults and children and obtained mixed results. Several putative pathogens, such as *Eubacterium*, *Porphyromonas*, and *Tannerella*, along with commensal lactobacilli and streptococci, positively associated with salivary cotinine concentrations in adults. Conversely, the abundances of other commensals such as *Rothia mucilaginosa*, and possibly beneficial taxa such as *S. parasanguinis,* were negatively associated with cotinine concentrations in adults. We note that cotinine concentration is important when studying the effects of smoking on the microbiome, and our associative results complement our comparisons of “smoker” to “nonsmoker.” Many of the above-mentioned genera and species have been shown to be enriched or depleted in similar ways, [(57, 63) and as reviewed in (64)], indicating there may be a dose-response to nicotine exposure. Cotinine concentrations only associated with three bacterial taxa in children (Lactobacilli and Streptococci that were unresolvable to species and somewhat uncommon), supporting our conclusion that ETS exposure has little effect on the child oral microbiome, possibly due children’s less direct exposure to ETS.

We assessed associations between microbial ASVs and levels of salivary adiponectin, CRP, and uric acid among children. Uric acid concentrations were inversely related to several ASVs with potentially pathogenic properties, such as *Porphyromonas pasteri* and *Prevotella pallens*, and the putative pathobiont *Parvimonas micra* (1, 65). This is interesting because uric acid may possess both pro- and antiinflammatory properties (66, 67). It may be that the inverse relationship between uric acid and certain taxa may indicate bacterial antiinflammatory activity or is evidence of an immune response to these bacteria. It should be noted that measuring inflammatory cytokines would assist in understanding the relationship and should be considered in future work. Previous work showed that high uric acid levels are associated with an altered oral microbiota, and together with our research, suggests an interaction between uric acid and oral microbes that may impact or indicate host health (68).

Additionally, we investigated the relationship between adiponectin and oral microbes, and found positive associations with ASVs of the commensal bacterium *Rothia mucilaginosa*, and a negative association with *S. parasanguinis* (a potentially beneficial species) (54). Higher adiponectin is generally antiinflammatory (69) and as oral infections are often inflammatory, adiponectin may be a useful biomeasure related to oral inflammation. Interestingly, we found only one significant association between salivary CRP concentrations and the oral microbiome (a *Stenotrophomonas* ASV). This was unexpected, as CRP is secreted by the gingiva (70), and has also been used as a marker for systemic inflammation and oral health in adults (22, 71), and chronic psychological stress in children (72). Most studies investigate links between adult oral health and CRP; however, this association may not be supported or particularly sensitive for younger individuals (73).

### Conclusion

Through a combination of biomeasure assays and 16S rRNA gene sequencing, our large-scale study illustrates that there are several underlying factors related to the human oral microbiome. We show that the oral microbial community is more similar within child/caregiver dyads, and that this microbiome potentially clusters into two “stomatotypes” largely driven by *Streptococcus* taxa. We also found that ETS exposure associates with certain oral microbes, and that the intensity of nicotine exposure likely correlates with more dramatic changes in adults. Lastly, we show that several microbial taxa may interact with salivary biomeasures related to metabolic activity, inflammation, and antioxidant potential, however more work is needed to understand the mechanisms underlying these associations and their relations to health. We suggest that future research investigate the complex interactions between host and microbe on a functional level, and that more large-scale studies are needed to understand the microbial ecology of oral health.

## Data Availability

Raw sequencing data are available on the NCBI Sequence Read Archive under BioProject accession number PRJNA689848, and code used to analyze the data is available on GitHub at github.com/jasonarothman/ECHO_90mo_microbiome. The complete ASV table has been uploaded to Zenodo and can be found at 10.5281/zenodo.7523443 (34).

## SUPPLEMENTAL MATERIAL

Figure S1 msystems.00036-23-s0001.pdf Alpha rarefaction analyses of the oral microbiome in children and caregivers.

ASM does not own the copyrights to Supplemental Material that may be linked to, or accessed through, an article. The authors have granted ASM a non-exclusive, world-wide license to publish the Supplemental Material files. Please contact the corresponding author directly for reuse.
