# Supplementary material for: Oral microbial communities in children, caregivers, and associations with salivary biomeasures and environmental tobacco smoke exposure
Source: mSystems. 2023 Jun 20;8(4):e00036-23. doi: 10.1128/msystems.00036-23 (PMC10470043; doi:10.1128/msystems.00036-23)
Supplement: Figure S1 — Alpha rarefaction analyses of the oral microbiome in children and caregivers. [file msystems.00036-23-s0001.pdf]

Supplemental information for: Oral microbial communities in children, caregivers, and associations with salivary biomeasures and environmental tobacco smoke exposure.

Jason A. Rothman[1,2#], Jenna L. Riis[2,3], Katrina R. Hamilton[2,7], Clancy Blair[4], Douglas A. Granger[2, 5-6], and Katrine L. Whiteson[1,2#]

[1]Department of Molecular Biology and Biochemistry, University of California, Irvine, Irvine, CA, 92697

[2]Institute for Interdisciplinary Salivary Bioscience Research, University of California, Irvine, CA, USA

[3]Department of Psychological Science, University of California, Irvine, CA, USA

[4]Department of Population Health and Department of Applied Psychology, New York University, New York, NY, USA

[5]Department of Acute and Chronic Care, Johns Hopkins University School of Nursing, and Department of Pediatrics Johns Hopkins University School of Medicine, Baltimore, MD, USA

[6]Salivary Bioscience Laboratory and Department of Psychology, University of Nebraska, Lincoln, NE, USA

[7]Department of Psychiatry and Behavioral Sciences, Johns Hopkins University School of Medicine, Baltimore, MD, USA

[#] **Co-corresponding authors**: Jason Rothman, Department of Molecular Biology and Biochemistry and Institute for Interdisciplinary Salivary Bioscience Research, University of California, Irvine, Irvine, CA, 92697, rothmanj@uci.edu, (949) 824-3509; Katrine Whiteson,

Department of Molecular Biology and Biochemistry and Institute for Interdisciplinary Salivary Bioscience Research, University of California, Irvine, Irvine, CA, 92697, katrine@uci.edu, (949) 824-9032

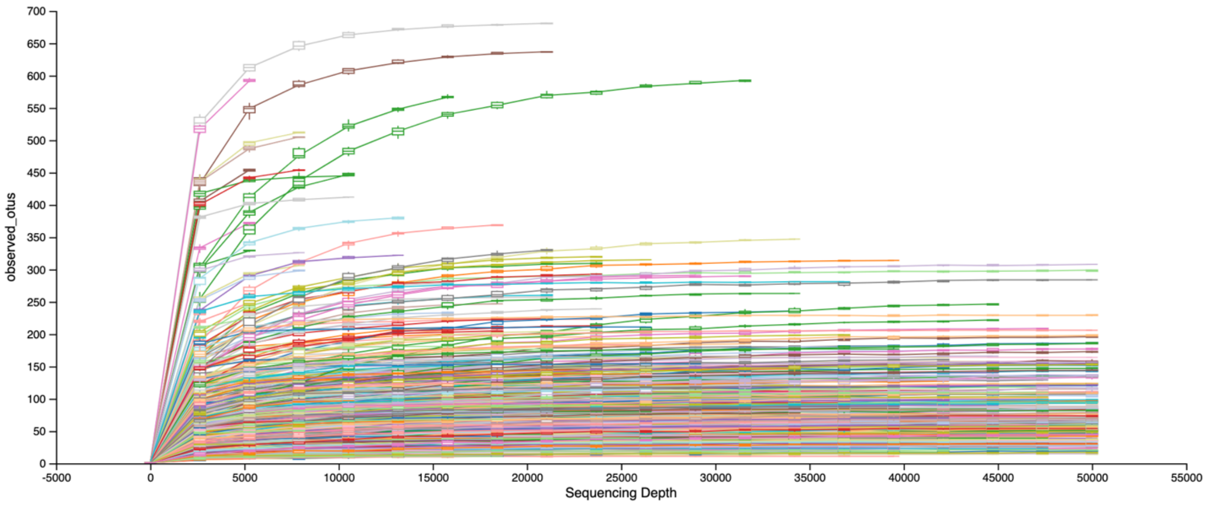

Fig. S1: Alpha rarefaction analyses of the oral microbiome in children and caregivers.
